# Baseline amyloid burden and global cognitive change during a home-based tDCS protocol in late-life depression: an exploratory single-arm study

**DOI:** 10.3389/fpsyt.2026.1916856

**Published:** 2026-09-03

**Authors:** Yiran Li, Jaesub Park, Jin Young Park, Woo Jung Kim

**Affiliations:** 1 Department of Psychiatry, Yongin Severance Hospital, Yongin, Gyeonggi, Republic of Korea; 2 Institute of Behavioral Science in Medicine, College of Medicine, Yonsei University, Seoul, Republic of Korea; 3 Yonsei Graduate Program in Cognitive Science, Yonsei University, Seoul, Republic of Korea; 4 Yonsei Beyond Lab, Yongin, Gyeonggi, Republic of Korea

**Keywords:** amyloid PET, cognitive impairment, geriatric depression scale, late-life depression, transcranial direct current stimulation

## Abstract

**Background:**

Late-life depression often co-occurs with cognitive difficulty, but whether amyloid burden is associated with mood and cognitive change during transcranial direct current stimulation (tDCS) in older adults is unknown.

**Methods:**

In this single-arm pre–post study, 41 older adults were enrolled; 3 withdrew before receiving the device, and 38 received home-based tDCS and completed baseline (V1), post-stimulation (V2), and follow-up (V3) assessments. Depression, insomnia, subjective cognition, MoCA, and a detailed cognitive battery were examined, and 31 change outcomes were regressed on Centiloid and 21 regional amyloid indicators with covariate adjustment.

**Results:**

No participant met the ≥50% GDS response criterion, whereas 60.5% showed a ≥2-point MoCA gain; the absent depression response reflects a floor effect expected from the low baseline severity rather than inefficacy. No association survived Benjamini–Hochberg correction across the complete exploratory family. In a biologically motivated Centiloid-only analysis, however, greater Centiloid burden was associated with smaller MoCA gain, and this association survived correction in both the full-analysis and per-protocol sets. Phonemic-fluency findings were less stable, and no comparable FDR-supported association was detected for depression, insomnia, or subjective cognition.

**Conclusions:**

The findings do not support a broad amyloid-related cognitive pattern, but they identify a focused, hypothesis-generating Centiloid–MoCA association during the protocol. Because the study was single-arm, neither the observed changes nor the amyloid association can be attributed to stimulation itself. Controlled studies with prespecified outcomes are required.

## Introduction

Late-life depression is common yet frequently underdiagnosed and undertreated, and in older adults it is closely intertwined with cognitive decline. Depressive symptoms often co-occur with cognitive difficulties, and the two domains influence each other bidirectionally: depression degrades attention and executive performance, while progressive cognitive decline can deepen hopelessness and withdrawal. Notably, late-life depression accompanied by cognitive impairment tends to respond more slowly and more variably to treatment (1). This entanglement of mood and cognition exposes the limits of treatments that target only one domain and motivates approaches that assess and address both. Recent comprehensive reviews summarize its clinical, pathophysiological, and therapeutic considerations (2).

Standard pharmacotherapy for late-life depression has important limitations. Relative to younger adults, older samples show smaller antidepressant effect sizes, higher placebo response, and delayed onset of action (1), while age-specific safety concerns (polypharmacy, drug–drug interactions, and anticholinergic burden) further constrain treatment. Barriers such as limited mobility, stigma, and geographic isolation compound these problems by impeding access to evidence-based care (3). Consequently, interest has grown in non-pharmacological neuromodulation that is well tolerated and accessible.

Transcranial direct current stimulation (tDCS) is a non-invasive technique that delivers weak direct current through scalp electrodes to modulate cortical excitability. Its antidepressant action is attributed to anodal stimulation of the left dorsolateral prefrontal cortex (DLPFC), restoring prefrontal hypoactivity, with neuroplastic effects that outlast the stimulation period (4). Its portability, low cost, and benign side-effect profile make it attractive for older and primary-care populations (5).

Evidence for tDCS efficacy in depression has accumulated through randomized controlled trials and their syntheses. Landmark trials compared tDCS with antidepressants (6, 7), and individual patient data meta-analyses and systematic reviews report that active tDCS significantly improves depression relative to sham (8–10). In a six-trial meta-analysis, active tDCS yielded higher response and remission rates than sham, with effect sizes comparable to antidepressants and repetitive transcranial magnetic stimulation and dropout rates that did not differ from sham; higher stimulation intensity predicted greater efficacy, whereas treatment resistance predicted poorer efficacy (8). tDCS has also been examined as an add-on for bipolar depression (11).

This evidence, however, derives largely from comparatively younger samples; data in older and cognitively impaired populations are sparser and less consistent. The geriatric evidence base has nonetheless strengthened. A systematic review and meta-analysis of 18 randomized trials in older adults (912 participants) reported a large pooled effect favoring active over sham stimulation for depressive symptoms (SMD = −0.96), although with substantial heterogeneity (13), whereas a smaller individual-participant analysis restricted to late-life depression remained inconclusive (12). That heterogeneity plausibly reflects clinical variability, comorbidity, and age- or neurodegeneration-related differences in cortical responsiveness. Less well characterized is the effect of tDCS on cognition in this population, and the biological factors that condition it. Because tDCS targets the prefrontal cortex, it may also affect cognition, particularly executive function and working memory. tDCS paired with cognitive training has influenced mood symptoms in older adults (14), and in a randomized clinical trial of older adults with remitted major depressive disorder or mild cognitive impairment, cognitive remediation combined with prefrontal tDCS slowed cognitive decline over several years of follow-up, with more prominent effects in those with remitted depression (15), and tDCS has been applied to cognition and mood in Alzheimer’s disease (16–20). Yet studies that jointly evaluate mood and cognitive change in older adults with depression and co-occurring cognitive difficulties remain scarce.

Finally, Alzheimer’s disease–related amyloid pathology is common in older adults and rises with age and APOE ϵ4 carriership (21, 22). Amyloid-β oligomers impair synaptic function and inhibit hippocampal long-term potentiation, a core substrate of neuroplasticity (23, 24). Because the after-effects of tDCS depend on comparable plasticity mechanisms, amyloid burden may plausibly attenuate cognitive responses to stimulation while leaving mood responses relatively unaffected. Although tDCS has been applied in Alzheimer’s disease (16, 17), few studies have examined whether baseline amyloid burden relates differently to post-tDCS change in mood versus cognition. Combining a harmonized global index (Centiloid) with regional standardized uptake value ratios (SUVR) allows this question to be examined at both global and regional levels.

Accordingly, in community-dwelling older adults with depression and co-occurring cognitive difficulties undergoing tDCS, the present single-arm pre–post study (a) describes pre–post change in depression (GDS) and cognition (MoCA and a detailed battery); (b) characterizes treatment response using literature-based GDS criteria; and (c) examines whether baseline amyloid PET (Centiloid and regional SUVR) is associated with change observed during the protocol.

## Methods

### Participants and design

Participants were recruited from community-dwelling older adults who presented with subjective depressive symptoms. Following an initial screening, a board-certified psychiatrist conducted clinical interviews to confirm the presence of clinically significant depression warranting treatment. A total of 41 older adults with depressive symptoms provided written consent and were enrolled. Eligibility was determined by a board-certified psychiatrist’s clinical diagnosis of clinically significant depression warranting treatment, established through a clinical interview; no minimum score on any depression rating scale was required for entry. Because screening scales such as the GDS have imperfect sensitivity in older adults and clinically evident depression can fall below the cutoff, enrollment was decided by the psychiatrist’s clinical interview rather than by a scale threshold. Cognitive impairment was not itself an inclusion criterion; participants were required only to have no prior diagnosis of dementia, and the MoCA was administered as a baseline cognitive screen rather than as an entry threshold. Individuals with a dementia diagnosis, current use of cognitive enhancers, delirium or other conditions that could cause cognitive impairment, or a history of intellectual disability were excluded. The cohort therefore comprised community-dwelling older adults with mild depression and preserved daily functioning, screened to exclude dementia, among whom co-occurring subjective and objective cognitive difficulty varied and was characterized at baseline. Cognitive status was not defined by a single cutoff: at screening, MoCA was used together with exclusion of dementia, cognitive-enhancer use, and other causes of cognitive impairment, and a detailed battery (MMSE, CERAD-K, and domain-specific tests) was administered at V1 and V3. Participants thus spanned the spectrum from subjective cognitive complaints to MCI-range performance, by design, while frank dementia was excluded.

This study was conducted as part of a larger project originally designed to investigate the neurobiological mechanisms of tDCS in older adults, with a particular focus on glymphatic system changes assessed via diffusion-tensor imaging analysis along the perivascular space before and after stimulation. To enable correlation analyses between MRI-derived glymphatic indices and clinical phenotypes, participants completed an extensive cognitive battery along with basic assessments of mood and sleep. Because the imaging analyses require additional processing and are still ongoing, MRI-derived measures are not included in the present report.

The current manuscript employed a single-arm, pre–post design with three assessment points (baseline before stimulation [V1], immediately after the stimulation course [V2], and at follow-up [V3]), and focuses on the clinical and cognitive outcomes of tDCS and on whether baseline amyloid status was associated with these outcomes. Of the 41 enrolled participants, 3 withdrew consent before receiving the device; the remaining 38 received home-based tDCS, completed all three assessments, and constituted the full analysis set. No clinically significant adverse effects were observed, and no participant discontinued treatment or reduced adherence because of adverse effects or tolerability problems.

Exclusion criteria were as follows: a diagnosis of dementia or current use of a cognitive enhancer (i.e., donepezil, rivastigmine, galantamine, or memantine); a history of intellectual disability; the presence of any other condition capable of producing cognitive impairment, such as delirium; a current alcohol use disorder; a past or current history of seizures or epilepsy; clinically significant cardiovascular, gastrointestinal, or respiratory disease; a history of brain surgery or cerebrovascular surgery such as carotid surgery; scalp deformity, inflammation, or other conditions impeding electrode attachment; any contraindication to the use of tDCS devices; severe visual or auditory impairment not correctable to an adequate level. Concomitant psychotropic medication status was recorded at screening. Ongoing antidepressant use was permitted to facilitate recruitment, provided the regimen had been stable, with no change in the antidepressant regimen, for at least 3 months before enrollment.

The study protocol was approved by the Institutional Review Board of Yongin Severance Hospital (IRB approval No.: 9-2024-0185), and the study was conducted in accordance with the Declaration of Helsinki. All participants provided written informed consent prior to participation. Recruitment was conducted between March 2025 and October 2025.

### Procedure

Assessments were organized across scheduled visits. At screening (S1), written informed consent was obtained, after which a psychiatrist conducted a clinical interview with each participant to determine eligibility. Demographic information was then collected. At baseline (V1), blood tests (including APOE genotyping), MRI (including DTI-ALPS), amyloid PET, and a neuropsychological battery were performed; the depression, cognition, and sleep scales were administered; and participants who proceeded to stimulation received tDCS training and were provided with the device. During the 4-week treatment period, participants self-administered home-based tDCS 5 to 7 days per week. At the post-intervention visit (V2), the device was returned, the same depression, cognition, and sleep scales were re-administered, and adverse events were assessed. At follow-up (V3), conducted 4 weeks after V2, participants underwent repeat MRI, blood testing, and a detailed neuropsychological battery, and the depression, cognition, and sleep scales were administered once more.

### tDCS intervention

This study employed a portable tDCS device (MINDD STIM+, Ybrain Inc., Seongnam, Republic of Korea) that has been approved by the Ministry of Food and Drug Safety (MFDS) of South Korea for at-home treatment of mild to moderate major depressive disorder (MDD). The anode was placed over the F3 region and the cathode over the F4 region, corresponding to the left and right dorsolateral prefrontal cortex (DLPFC), respectively, based on the International 10–20 system for electroencephalography. A dedicated headband was used to fix electrode position and ensure consistent placement across sessions. Each session delivered an individually optimized direct current of 1.5–2 mA for 30 minutes, and 5 to 7 sessions per week were prescribed over a total of 4 weeks. The F3-anodal/F4-cathodal bifrontal montage was the standard configuration of the MFDS-approved device and corresponds to the target used in landmark antidepressant tDCS trials, in which anodal stimulation of the left DLPFC (with the cathode over the right DLPFC) increases left- while reducing right-prefrontal excitability (4, 6, 7). This target was also pertinent to the cognitive outcomes examined here: the DLPFC is a core node of the frontoparietal (central-executive) network that subserves working memory and executive function (25), and in late-life depression this region shows hypoactivation and reduced cognitive-control connectivity linked to both depressive and executive dysfunction (26). Consistency of electrode positioning across the home-based sessions was supported by a dedicated headband that held the anode (F3) and cathode (F4) at the prescribed left- and right-DLPFC positions, together with structured pre-treatment training in self-application and adherence to the prescribing physician’s guidance.

## Measures

### Depression

Depressive symptoms were assessed with the 30-item Geriatric Depression Scale (GDS) (27), a self-report screening measure with a yes/no response format and a total score ranging from 0 to 30, where higher scores indicate greater depression. Scores of 0–10 are conventionally treated as the normal range, and a cutoff of 11 indicates suspected depression (27, 28). The GDS was administered at V1, V2, and V3.

Although the GDS is well established as a continuous severity measure, the use of categorical response thresholds on the GDS rests on a thin evidence base, with only a handful of precedents (1, 3, 29); we therefore adopted literature-based thresholds and report categorical outcomes as descriptive (see Data Analysis).

### Insomnia and subjective cognition

Sleep disturbance was measured with the Insomnia Severity Index (ISI) (30), a 7-item self-report scale (total 0–28) on which higher scores reflect more severe insomnia. Subjective cognitive complaints were assessed with a subjective cognitive decline questionnaire (SCD-Q) (31), with higher scores indicating greater perceived cognitive difficulty. Both were administered at V1, V2, and V3.

### Global cognition

Global cognitive status was screened with the Montreal Cognitive Assessment (MoCA; total 0–30), a measure sensitive to MCI on which higher scores indicate better performance, administered at all three timepoints. A MoCA increase of ≥2 points from baseline was used to define cognitive response.

### Detailed neuropsychological battery

At V1 and V3, a detailed battery was administered, comprising the Mini-Mental State Examination (MMSE) and CERAD-K total scores; orientation (time and place) and attention; language (Boston Naming Test and verbal/semantic fluency); verbal memory (word-list immediate recall, delayed recall, and recognition); constructional praxis and constructional recall; processing speed and set-shifting (Trail Making Test A and B; Stroop); and the Frontal Assessment Battery (FAB) with its subtests (similarities [conceptualization], phonemic [lexical] fluency, motor series, conflicting instructions, go/no-go, and prehension) and the FAB total.

### Amyloid PET and APOE genotyping

Baseline cerebral amyloid burden was quantified from [^18^F]flutemetamol positron emission tomography on the harmonized Centiloid scale (on which 0 anchors the level of amyloid-negative young controls and 100 the typical level in Alzheimer’s disease), together with 21 regional standardized uptake value ratios (SUVRs) spanning frontal, parietal, temporal, precuneus, cingulate, and striatal regions and a global region of interest, each expressed as left, right, and bilateral values. Centiloid quantification was performed using SCALE PET 2.0 (v2.0.1, Neurophet Inc., Seoul, Republic of Korea), which provides fully automated Centiloid quantification from PET images. SCALE PET requires a 3D T1-weighted MRI acquired at 1 mm slice thickness: each participant’s MR image was automatically segmented to define cortical regions of interest in individual space, after which the PET image was co-registered to the MRI to compute the Centiloid value without user inspection. SCALE PET 2.0 has been approved by the MFDS. APOE ϵ4 carrier status (carrier vs. non-carrier) was determined by genotyping and entered as a covariate.

### Data analysis

Analyses were conducted in Stata using heteroscedasticity-robust standard errors. The detailed neuropsychological battery was administered at V1 and V3, so change in those domains was computed as the V1-to-V3 difference. The analysis proceeded in five parts. All analyses were performed on two sets: the full analysis set (FA), comprising all participants who received at least one stimulation session and had a valid follow-up assessment (N = 38), and a per-protocol set (PP), restricted to participants who completed at least 80% of the prescribed sessions (≥16 of the planned 20–28). The PP set excluded four participants who completed 2, 7, 11, and 11 sessions, respectively, owing to difficulty self-operating the device; none of these exclusions reflected adverse events or tolerability problems (N = 34). The FA was the primary set; the PP analysis is reported alongside it as a sensitivity check, and the two sets yielded consistent results. This analytic framing follows prior home-based tDCS trials from our group, which adopted an intention-to-treat or full-analysis-set primary analysis with a completer (per-protocol) sensitivity analysis (32–34).

First, baseline characteristics were summarized descriptively. Second, timepoint means (V1, V2, V3) for the four core outcomes were tabulated; within-person change from baseline was tested with paired-samples *t* tests (or Wilcoxon signed-rank tests) accompanied by Cohen’s *d*_z_ effect sizes; in addition, the overall time effect across V1, V2, and V3 was evaluated with linear mixed models (random intercept) adjusting for age, sex, education, APOE ϵ4, and antidepressant use (Wald test).

Third, proportions meeting prespecified thresholds were computed with Wilson 95% confidence intervals: a ≥30% reduction (1) and a ≥50% reduction (3, 29); cognitive response was defined as a MoCA increase of ≥2 points. Because the sample’s mean baseline GDS was close to the remission cutoff (≤10), remission could not be meaningfully evaluated and is not analyzed further; analyses therefore focus on response.

For the categorical analyses, a participant was classified as a depression responder if the GDS total decreased by at least the criterion percentage from baseline to the index timepoint (V2 or V3), and as a non-responder otherwise, using two thresholds: the conventional ≥50% reduction (3, 8, 29) and a more lenient ≥30% reduction appropriate to a self-report screening scale (1). To avoid circularity, responders and non-responders were never compared on the change score that defined them; any responder/non-responder contrast was confined to logically independent variables (baseline characteristics, amyloid burden, APOE ϵ4, and the detailed cognitive domains) and was interpreted descriptively given the single-arm design.

Fourth, to test whether baseline amyloid was associated with change observed during the protocol, covariate-adjusted linear regressions were fit in which each protocol-period change outcome (31 outcomes) was regressed separately on each amyloid indicator (the Centiloid index plus 21 regional SUVRs); all models adjusted for age, sex, education, APOE ϵ4, and antidepressant use, with the amyloid coefficient reported as a standardized β, its *p* value, and model *R²*. Because the analysis comprised many uncorrected tests, these results are exploratory and hypothesis-generating.

Fifth, logistic regressions adjusting for age, sex, education, APOE ϵ4, and antidepressant use modeled responder status as a function of amyloid (Centiloid and regional SUVR), reporting odds ratios; regional and V2 models exhibited severe quasi-separation given few events, so interpretation was confined to the Centiloid models. As a single-arm study, the design cannot separate treatment effects from natural course, regression to the mean, or retest effects; accordingly, response rates are descriptive rather than efficacy estimates. All analyses used complete cases (n = 38); the 3 who withdrew before receiving the device contributed no data.

## Results

**Figure 1:**
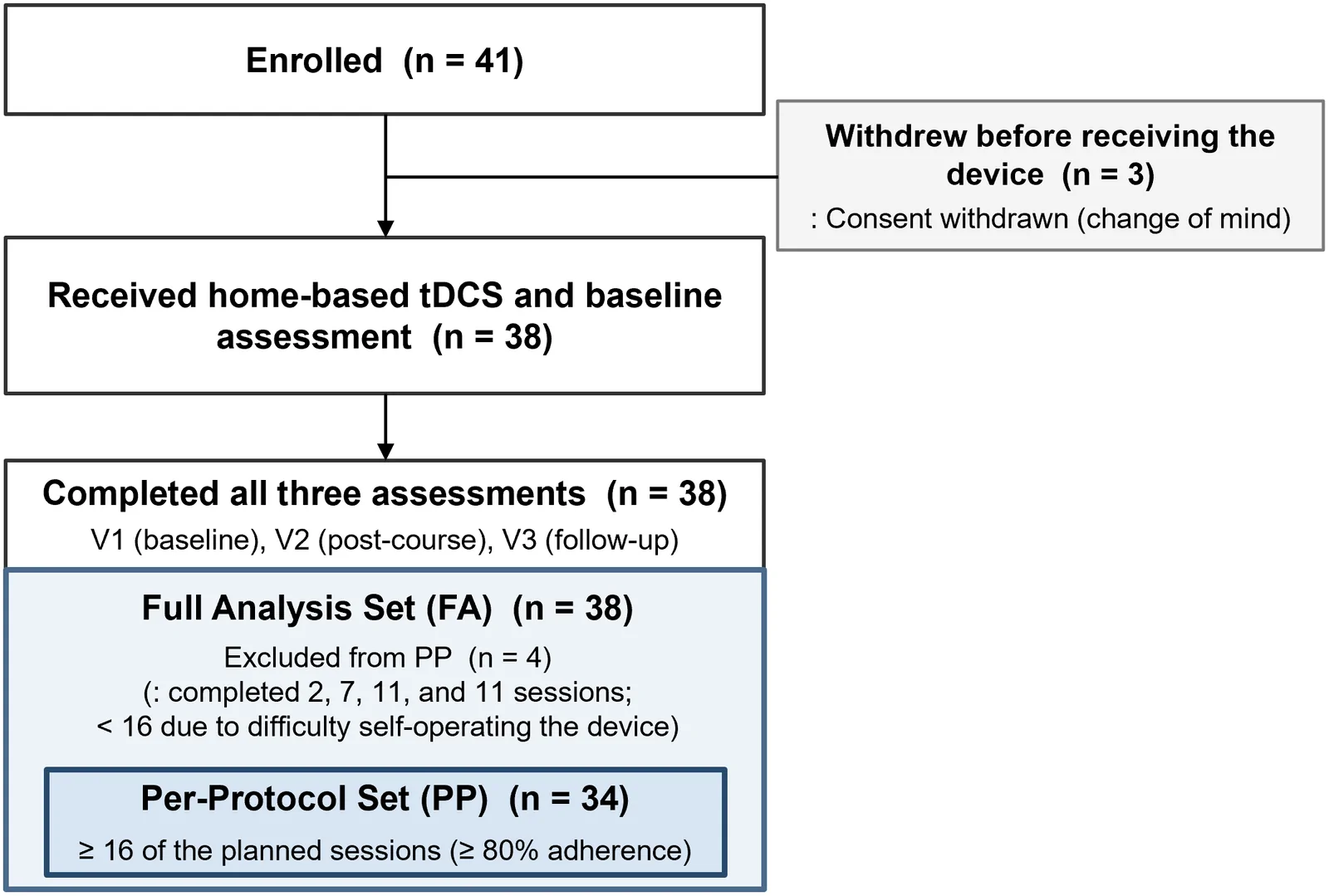
Participant flow. CONSORT-style participant flow. Of 41 enrolled, 3 withdrew consent before receiving the device; the remaining 38 received home-based tDCS and completed all three assessments (full analysis set). Thirty-four met the per-protocol criterion of completing at least 16 of the planned 20–28 sessions (≥80% adherence). Four participants were excluded from the per-protocol set after completing 2, 7, 11, and 11 sessions, respectively, owing to difficulty self-operating the device; none of these exclusions reflected adverse events or tolerability problems. No clinically significant adverse effects were observed, and no participant discontinued treatment or reduced adherence because of adverse effects.

This single-arm pre–post (V1→V3) study enrolled 41 participants, of whom 3 withdrew consent before receiving the device and 38 completed all three assessments (**Figure 1**). Unless otherwise specified, adjusted analyses included age, sex, education, APOE ϵ4, and antidepressant use as covariates.

### Baseline characteristics

**Table 1** summarizes the baseline demographic and clinical characteristics of the 38 completers. The sample was, on average, 68.0 years old with approximately 11 years of education, and 18.4% (7/38) carried APOE ϵ4. Over the treatment course, participants received a mean of 24.3 stimulation sessions (SD = 6.2; range 2–28). Clinically, the mean baseline GDS of 12.79 sat just above the suspected-depression cutoff (≥11), indicating mild depressive symptomatology with limited room for proportional improvement, and the mean MoCA of 23.71 was consistent with the MCI range. The mean baseline Centiloid was 7.53, within the normal-to-borderline range, but the large standard deviation (SD = 30.75) and wide range (−21.9 to 110.8) indicate that the sample spanned both amyloid-negative and clearly amyloid-positive participants, a heterogeneity that is informative for the amyloid-prediction analyses reported below. At enrollment, 8 of the 38 participants (21.1%) were taking a stable antidepressant: escitalopram in four, agomelatine and vortioxetine in two each, and sertraline and mirtazapine in one each (two participants were on two agents). Six had maintained an unchanged regimen for approximately 1.0 to 3.5 years, whereas two had had an antidepressant added shortly before the 3-month stability window (**Supplementary Table S1**).

**Table 1:** Baseline demographic and clinical characteristics.

Characteristic	FA (N = 38)	PP (N = 34)
Age (years)	68.0 (5.1)	68.2 (5.1)
Sex, female, n (%)	33 (86.8)	30 (88.2)
Education (years)	11.1 (3.0)	10.9 (3.0)
APOE ϵ4 carrier, n (%)	7 (18.4)	6 (17.6)
tDCS sessions	24.3 (6.2)	26.2 (2.1)
Baseline Centiloid	7.53 (30.75)	9.32 (32.03)
Baseline GDS	12.79 (3.58)	12.47 (3.31)
Baseline ISI	13.00 (6.35)	13.21 (6.09)
Baseline SCD-Q	8.76 (4.56)	8.44 (4.63)
Baseline MoCA	23.71 (3.27)	23.68 (3.42)

Enrolled N = 41; 3 withdrew consent before receiving the device. APOE ϵ4, Centiloid, and tDCS sessions n = 38. FA = full analysis set (N = 38); PP = per-protocol set (N = 34), restricted to participants completing ≥16 of the planned 20–28 sessions. Values are M (SD) or n (%).

### Change over time in primary outcomes

**Figure 2:**
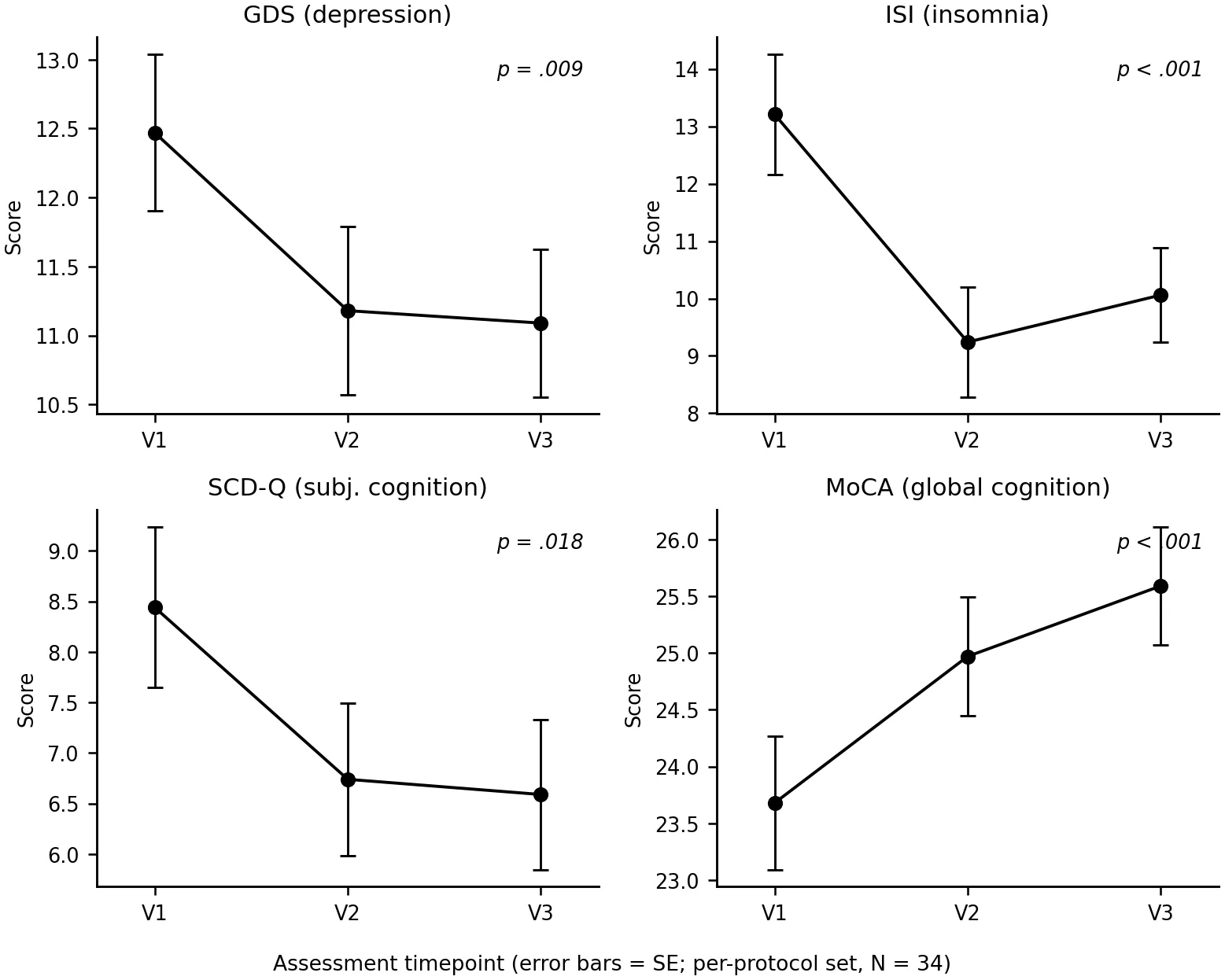
Change in clinical and cognitive outcomes across timepoints. Mean scores at baseline (V1), post-stimulation (V2), and follow-up (V3) for the per-protocol set (N = 34). Error bars represent standard errors. GDS, Geriatric Depression Scale; ISI, Insomnia Severity Index; SCD-Q, Subjective Cognitive Decline Questionnaire; MoCA, Montreal Cognitive Assessment. p values are for the overall time effect from linear mixed models.

Depression (GDS) declined modestly and monotonically (12.79 → 11.63 → 11.39), a small absolute change consistent with the low, near-cutoff baseline. Insomnia (ISI) showed the largest early improvement, falling from 13.00 at baseline to 8.95 immediately post-stimulation before partially rebounding at follow-up (9.89). Subjective cognition (SCD-Q) decreased steadily across the three timepoints (8.76 → 7.42 → 7.18). Objective cognition (MoCA) rose monotonically and exhibited the most pronounced change (23.71 → 24.97 → 25.66), an increase of approximately 1.9 points from baseline to follow-up. The overall time effect, tested with linear mixed models (**Table 2**), was significant for GDS, ISI, and MoCA, and reached significance for SCD-Q in the per-protocol set; effects were consistent across the full analysis and per-protocol sets (**Figure 2**).

**Table 2:** Descriptive statistics for primary outcomes by timepoint.

Outcome	Set	V1 (baseline)	V2	V3	Wald χ² (time)	p
GDS	FA	12.79 (3.58)	11.63 (3.91)	11.39 (3.45)	10.67	.005
	PP	12.47 (3.31)	11.18 (3.57)	11.09 (3.12)	9.51	.009
ISI	FA	13.00 (6.35)	8.95 (5.77)	9.89 (5.11)	18.52	<.001
	PP	13.21 (6.09)	9.24 (5.57)	10.06 (4.77)	16.37	<.001
SCD-Q	FA	8.76 (4.56)	7.42 (5.06)	7.18 (4.47)	5.89	.053
	PP	8.44 (4.63)	6.74 (4.41)	6.59 (4.33)	7.99	.018
MoCA	FA	23.71 (3.27)	24.97 (3.02)	25.66 (2.90)	31.22	<.001
	PP	23.68 (3.42)	24.97 (3.03)	25.59 (3.02)	26.16	<.001

Values are mean (SD). The time effect is the Wald test of the timepoint factor from a linear mixed model with a random intercept, adjusting for age, sex, education, APOE ϵ4, and antidepressant use. df = 2 for each time effect. Results are shown separately for the full analysis set (FA, N = 38) and the per-protocol set (PP, N = 34); the time effect was significant in both sets for all outcomes except SCD-Q, which reached significance in the PP set only.

These trajectories can also be summarized as categorical response rates. The proportions meeting each response criterion (with Wilson 95% confidence intervals) showed the same pattern (**Supplementary Table S2**). The depression results were dominated by a floor effect: at follow-up, no participant achieved the conventional ≥50% reduction (0/38, 0%) and only 10.5% (4/38) achieved a ≥30% reduction, reflecting that many participants began near the normal range. The post-stimulation (V2) figures were broadly similar, with a slightly higher ≥30% response rate (18.4%). In sharp contrast, cognitive response (MoCA ≥2-point gain) was common at both timepoints, met by 47.4% (18/38) immediately after stimulation (V2) and rising to 60.5% (23/38) at follow-up (V3). The juxtaposition of a 0% depression response and a 60.5% cognitive response at follow-up constitutes the study’s central descriptive finding: a marked dissociation between mood and cognitive outcomes.

To complement the continuous-change analyses, baseline Centiloid was used to predict each binary response outcome in covariate-adjusted logistic regressions (adjusting for age, sex, education, APOE ϵ4, and antidepressant use). For the cognitive response, higher baseline amyloid was associated with lower odds of achieving a MoCA ≥2-point gain, and this negative association strengthened from the immediate post-stimulation timepoint to follow-up (V2: OR = 1.00 per Centiloid unit, p = .920; V3: OR = 0.91, p = .112), mirroring the continuous-change finding that higher amyloid was associated with smaller cognitive gain; however, neither reached statistical significance in this small sample. No Centiloid model for any depression response criterion reached significance, and the regional and several V2 models showed severe quasi-separation given the few events, so those estimates are not interpreted.

### Baseline amyloid PET and protocol-period change

Baseline amyloid was examined as a predictor of protocol-period change. Each change outcome was regressed on amyloid (Centiloid and regional SUVRs), adjusting for age, sex, education, APOE ϵ4, and antidepressant use; **Table 3** reports the key nominal models. No statistically detectable association was observed for change in insomnia (ISI) or subjective cognition (SCD-Q), the strongest being ISI change on precuneus SUVR (β = 0.298, p = .086). Most depression models were likewise non-significant in the full analysis set, including GDS change on frontal SUVR (β = 0.129, p = .196), although the corresponding per-protocol model was nominally significant (β = 0.220, p = .047) without surviving correction (q = .560). The Centiloid–GDS model was non-significant in the full analysis set (β = 0.193, p = .071) and only nominally significant in the per-protocol set (β = 0.259, p = .041); it did not survive correction (q = .440 and .267). Two features qualify this model. The coefficient is positive, so the association is not one of greater amyloid predicting more severe depression but of greater amyloid predicting a smaller reduction in GDS score over the protocol period. Significance also emerged only in the per-protocol set, which comprises adherent completers and is more homogeneous in exposure; the coefficient in the full analysis set ran in the same direction with a comparable effect size, indicating that the difference between sets reflects precision and sample composition rather than a distinct effect. These null and nominal affective findings do not establish absence of an amyloid–mood relationship, particularly given the small sample and restricted range of baseline depression severity, but they provide no multiplicity-controlled support for one in this dataset.

**Table 3:** Baseline amyloid PET and protocol-period change.

Outcome (change, V1–V3)	Set	PET predictor	n	B	SE	β	p	R²
MoCA	PP	Centiloid	34	−0.027	0.006	−0.423	<.001	.560
	FA	Centiloid	38	−0.028	0.007	−0.448	<.001	.520
FAB phonemic fluency	PP	Centiloid	34	−0.010	0.004	−0.404	.013	.294
	FA	Centiloid	38	−0.010	0.003	−0.402	.006	.249
FAB phonemic fluency	PP	Cingulate SUVR	34	−1.970	0.719	−0.444	.011	.311
	FA	Cingulate SUVR	38	−2.058	0.627	−0.454	.003	.284
FAB similarities	PP	Centiloid	34	0.009	0.004	0.517	.017	.322
	FA	Centiloid	38	0.008	0.004	0.434	.037	.246
GDS	PP	Frontal SUVR	34	3.554	1.711	0.220	.047	.342
	FA	Frontal SUVR	38	2.062	1.559	0.129	.196	.291
ISI	PP	Precuneus SUVR	34	10.728	6.055	0.338	.088	.141
	FA	Precuneus SUVR	38	9.348	5.266	0.298	.086	.130

**Figure 3:**
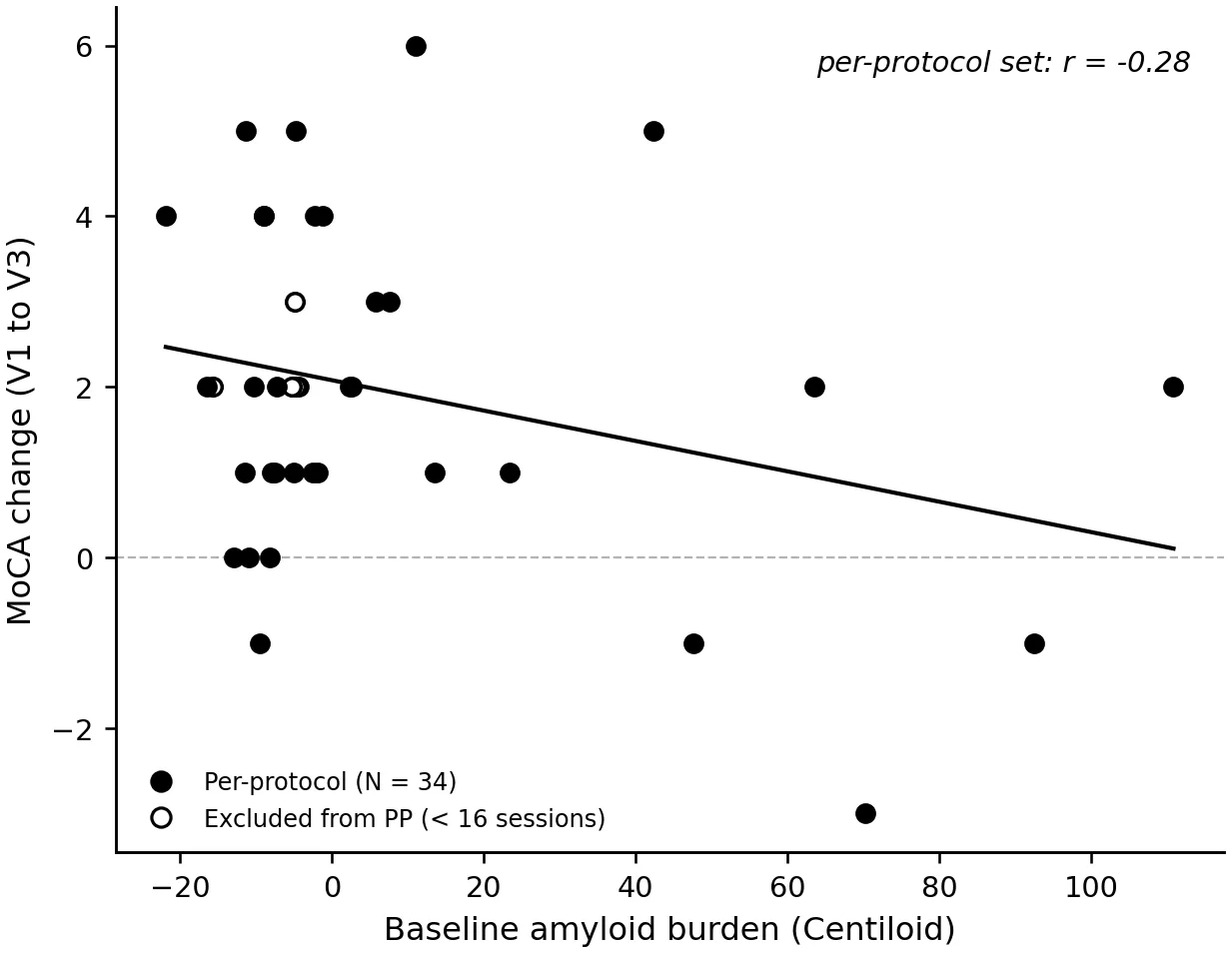
Baseline amyloid burden and change in global cognition. Each point represents one participant. Filled circles denote the per-protocol set (N = 34); open circles denote participants excluded from the per-protocol set for completing fewer than 16 sessions. The line is the ordinary least-squares fit in the per-protocol set (r = −.28). Higher baseline amyloid burden was associated with smaller gains in global cognition (MoCA). Centiloid, standardized amyloid PET scale; MoCA, Montreal Cognitive Assessment.

Adjusted for age, sex, education, APOE ϵ4, and antidepressant use. n denotes the set-specific analytic sample (PP = 34; FA = 38). The table reports raw p values for selected exploratory models, estimated with heteroskedasticity-robust standard errors. Positive coefficients for GDS and ISI indicate that higher baseline amyloid was associated with a smaller reduction in symptom score, not with greater baseline severity. Benjamini–Hochberg adjusted results for all 682 planned models per set are provided in **Supplementary Tables S3** and **S4**. R² values are from the full covariate-adjusted model; **Figure 3** shows the unadjusted bivariate association between baseline amyloid and MoCA change (r = −.28). Estimates are shown for the per-protocol (PP) set followed by the full analysis (FA) set; FA was the primary set and PP a sensitivity set.

The most statistically stable signal involved global cognition. MoCA change was inversely associated with Centiloid in both analysis sets (FA β = −0.448, p <.001; PP β = −0.423, p <.001) (**Figure 3**). Phonemic-fluency change also showed negative associations with Centiloid and cingulate SUVR, but this signal was less robust because it did not survive the per-protocol restriction after outcome-specific correction. One isolated association ran in the opposite direction: FAB similarities increased with higher Centiloid (β = 0.434, p = .020). Taken together, the results do not support a broad or convergent amyloid–cognition pattern. Rather, they identify a focused global-cognition signal, with phonemic fluency as secondary and less stable evidence and FAB similarities as a provisional, directionally discordant finding.

To quantify multiplicity directly, we estimated all 31 change outcomes by 22 amyloid indicators within each analysis set (682 models per set; 660 estimable because FAB prehension showed no variance) and applied the Benjamini–Hochberg procedure under complementary family definitions (**Supplementary Table S4**). Thirty-one models in the full analysis set and 32 in the per-protocol set were nominally significant at p <.05, close to the rough chance benchmark of approximately 33 of 660; because both outcomes and amyloid indicators are correlated, this count is informative but not an exact binomial test. No association survived correction when all estimable models were treated as one global exploratory family (minimum q = .120 and .061). In the biologically motivated Centiloid-only family across the 31 outcomes, however, MoCA change remained significant in both sets (q = .005 and .003), whereas phonemic fluency, depression, MMSE, and FAB similarities did not (q ≥ .088). Within the phonemic-fluency family across the 22 highly correlated amyloid indicators, all indicators survived correction in the full analysis set (q = .017 to .029) but none did in the per-protocol set (q = .069 to .100). Because a large number of models was estimated relative to the sample size, these exploratory analyses are best interpreted from the consistency of the overall pattern across outcomes and amyloid indicators rather than from individual p-values, and the corrections reported here were undertaken to characterize that pattern rather than to adjudicate single models. Read in that way, the pattern is concentrated rather than diffuse: the global-family analysis argues against a broad amyloid-related effect across cognition, whereas the Centiloid-only analysis preserves a limited and specific MoCA hypothesis that requires prospective confirmation.

## Discussion

In older adults with depression and co-occurring cognitive difficulties, this single-arm study yielded three findings. First, change over the protocol was asymmetric across domains: global cognition rose by approximately 1.9 MoCA points (23.71 → 25.66), whereas depression fell by about 1.4 GDS points (12.79 → 11.39). Second, the asymmetry was sharper categorically: no participant met the conventional ≥50% GDS reduction criterion, whereas 55.9% of the per-protocol set and 60.5% of the full-analysis set gained ≥2 MoCA points. Third, the amyloid signal was concentrated rather than diffuse. The inverse Centiloid–MoCA association was the clearest result and survived correction within the biologically motivated Centiloid-only family in both analysis sets, whereas no multiplicity-controlled association emerged for depression, insomnia, or subjective cognition. The data thus point to a focused global-cognition hypothesis.

The complete absence of ≥50% depression responders is an arithmetic consequence of baseline severity rather than evidence of inefficacy. With a mean baseline GDS of 12.79, barely above the screening cutoff of 11, a 50% reduction would require reaching approximately 6, deep within the asymptomatic range, which few mildly symptomatic older adults attain. In a comparable multicenter trial of cognitive behavioral therapy for late-life depression that also used the 30-item GDS (29), a higher baseline severity (M ≈ 20.7) yielded a 36–40% response, confirming that the present low rate reflects a floor rather than a failure.

The concentration of the strongest amyloid signal in global cognition, with no comparable corrected signal for affective or subjective outcomes, has a coherent biological reading. The limbic and prefrontal–striatal circuits implicated in late-life depression (25, 26) are anatomically distinct from the temporoparietal and default-mode regions in which fibrillar amyloid accumulates earliest (35–37). This separation aligns with converging evidence that, in older adults, amyloid burden tracks cognitive rather than affective status: depressive symptoms have been linked to tau rather than amyloid on positron emission tomography (38), and meta-analytic estimates of the amyloid–depression association are near null (39). Global cognition, by contrast, is more sensitive to the synaptic and network-level consequences of amyloid, given that amyloid-β oligomers impair long-term potentiation and synaptic plasticity (23, 24) and that elevated amyloid predicts subsequent cognitive decline even in non-demented older adults (40). A complementary methodological reading is also plausible: the modest baseline depression severity left little variance for proportional mood improvement, reducing power to detect amyloid-related differences, consistent with evidence that the detectable magnitude of treatment-related change diminishes at lower baseline severity (41, 42). These readings are not mutually exclusive, and the absence of an FDR-supported affective association is best read as no statistically detectable relationship in this sample rather than as evidence of none.

These results extend a literature dominated by younger, more severely depressed samples. The antidepressant signal in tDCS meta-analyses (response ≈ 34%) (8–10) was obtained where baseline severity left room for ≥50% change, and the present mildly depressed geriatric sample illustrates the boundary conditions of that criterion. The cognitive gains accord with reports that prefrontal tDCS can influence cognition in older and neurodegenerative populations (14, 16, 17), and the amyloid finding adds a biological qualifier those studies did not test: cognitive change observed during a stimulation protocol may be graded by underlying pathology.

If replicated, the focused Centiloid–MoCA association would connect these findings to a broader precision-neuromodulation framework, in which pretreatment biology shapes the magnitude of observed change. Response to non-invasive brain stimulation varies substantially across individuals, and factors beyond clinical presentation, including baseline cortical excitability, the capacity for stimulation-induced plasticity, and the integrity of targeted functional networks, have been proposed as contributors (43–45). Connectivity-based targeting has emerged as one individualizing principle in depression (43, 46), and motor-evoked potentials and TMS-EEG measures have been examined as candidate predictors across rTMS and tDCS (47, 48). Within this framework, baseline amyloid would be one potential component of a multimodal model rather than a stand-alone marker, and future studies could test whether Centiloid adds predictive information beyond cortical excitability, physiological plasticity, and network integrity (23, 24).

These findings are hypothesis-generating, and the considerations below bound how widely they generalize rather than whether the central pattern holds within this cohort. That pattern was consistent: the inverse Centiloid–MoCA association was stable across both the full-analysis and per-protocol sets and was the one association to survive correction within the biologically motivated Centiloid-only family, a convergence more readily explained by a genuine relationship than by chance. Because many models were estimated relative to the sample size, this signal is best weighed from the consistency of the overall pattern rather than from any individual p-value; on the same principle, the remaining associations, and the categorical response analyses whose event counts were low, mark targets for confirmation rather than established null results. APOE ϵ4 was entered as a covariate, so the amyloid–cognition association appears at least partly ϵ4-independent (21, 22), although with only seven carriers a formal amyloid-by-APOE interaction was not estimable, and ϵ4 may also act through amyloid-independent pathways relevant to stimulation response (49). Depression was established by clinical interview and cognitive status characterized dimensionally, which reflects the mixed presentations of geriatric practice while qualifying generalization to any single diagnostic group.

Two internal checks argue against trivial explanations of the cognitive findings. Treatment exposure varied, yet the number of completed sessions did not predict MoCA change (B = 0.04, SE = 0.17, p = .81), the amyloid coefficient retained its negative direction (B = −0.018, p = .11), and restricting the sample to adherent completers left the association essentially unchanged. A single-arm design cannot by itself separate protocol-related change from retest effects or other longitudinal factors, so the observed gains are not presented as an effect of stimulation per se; the amyloid gradient, however, sits awkwardly with a simple retest account, which would not predict that cognitive gains scale with baseline pathology. What the design does establish is specific and testable: in a well-characterized geriatric cohort, baseline amyloid burden tracked the magnitude of cognitive change more closely than affective change over the protocol, a relationship now defined precisely enough to be tested directly in a sham-controlled trial.

### Clinical implications

At a broad level, the parallel change in mood and cognition, more marked for cognition, is compatible with a dual-domain pattern of change in late-life depression accompanied by cognitive complaints, though the single-arm design leaves this a hypothesis rather than a demonstrated stimulation effect. The data do not justify using amyloid status to decide who should or should not receive tDCS. Their value is narrower: to prioritize a testable hypothesis that global amyloid burden, indexed by Centiloid, may be associated with global cognitive change during a tDCS protocol. Future sham-controlled trials should prespecify MoCA or another global-cognition endpoint, limit the number of amyloid hypotheses, and test whether the association is specific to active stimulation.

## Conclusion

During a home-based tDCS protocol in older adults with depression and co-occurring cognitive difficulties, mood and cognition changed in parallel, with larger gains for cognition. Higher baseline amyloid burden was associated with smaller gains in global cognition, whereas the affective associations were nominal only. Because the study was single-arm, these changes cannot be attributed to stimulation itself; controlled evidence for the intervention in older adults is nonetheless now reasonably well established (13). The study’s contribution is to nominate baseline amyloid as a candidate marker of cognitive change observed during the protocol and to provide a literature-grounded template for defining GDS response in mildly depressed older adults. Adequately powered, sham-controlled trials can test directly whether baseline amyloid moderates treatment-related cognitive response.

## Data Availability

The original contributions presented in the study are included in the article/**Supplementary Material**. Further inquiries can be directed to the corresponding author.

## Supplementary material

Supplementary Table S1 Antidepressant regimens of the eight participants receiving a stable antidepressant at enrollment.

Supplementary Table S2 Treatment response frequencies and baseline Centiloid logistic prediction at V2 and V3.

Supplementary Table S3 Amyloid prediction of treatment-related change across all 31 outcomes.

Supplementary Table S4 Benjamini-Hochberg false discovery rate correction for the exploratory amyloid analyses (31 change outcomes x 22 amyloid indicators).
