# Supplementary material for: Baseline amyloid burden and global cognitive change during a home-based tDCS protocol in late-life depression: an exploratory single-arm study
Source: Front Psychiatry. 2026 Sep 3;17:1916856. doi: 10.3389/fpsyt.2026.1916856 (PMC13582536; doi:10.3389/fpsyt.2026.1916856)
Supplement: Supplementary file 1 [file SupplementaryFile1.pdf]

Supplementary Material

Supplementary Table S1. Antidepressant regimens of the eight participants receiving a stable antidepressant at enrollment.

Participant	Antidepressant (generic)	No. of agents	Regimen status
P1	escitalopram	1	Unchanged ~1.0–3.5 yr
P2	sertraline	1	Unchanged ~1.0–3.5 yr
P3	mirtazapine; escitalopram	2	Escitalopram added just before the 3-month window
P4	agomelatine	1	Unchanged ~1.0–3.5 yr
P5	vortioxetine	1	Unchanged ~1.0–3.5 yr
P6	vortioxetine	1	Unchanged ~1.0–3.5 yr
P7	agomelatine; escitalopram	2	Agomelatine added just before the 3-month window
P8	escitalopram	1	Unchanged ~1.0–3.5 yr

Note. Antidepressants are listed by active ingredient (generic name). Two participants (P3, P7) had an antidepressant added just before the 3-month pre-enrollment stability window; the other six had unchanged regimens for approximately 1.0 to 3.5 years. Amounts are not reported because only antidepressant use (yes/no) was modeled as a covariate.

Supplementary Table S2. Treatment response frequencies and baseline Centiloid logistic prediction at V2 and V3.

Outcome	Set	Timepoint	n/N	%	95% CI	OR (Centiloid)	p
GDS response: ≥30% reduction	PP	V2	7/34	20.6	[10.4, 36.8]	1.023	.140
	FA	V2	7/38	18.4	[9.2, 33.4]	1.025	.114
GDS response: ≥30% reduction	PP	V3	4/34	11.8	[4.7, 26.6]	0.961	.264
	FA	V3	4/38	10.5	[4.2, 24.1]	0.971	.347
GDS response: ≥50% reduction	PP	V2	1/34	2.9	[0.5, 14.9]	1.224	.856
	FA	V2	1/38	2.6	[0.5, 13.5]	1.224	.856
GDS response: ≥50% reduction	PP	V3	0/34	0.0	[0.0, 10.2]	—	—
	FA	V3	0/38	0.0	[0.0, 9.2]	—	—
MoCA response: ≥2-point gain	PP	V2	16/34	47.1	[31.5, 63.3]	1.001	.923
	FA	V2	18/38	47.4	[32.5, 62.7]	1.001	.920
MoCA response: ≥2-point gain	PP	V3	19/34	55.9	[39.5, 71.1]	0.942	.235
	FA	V3	23/38	60.5	[44.7, 74.4]	0.909	.112

Note. n/N is the number meeting each criterion out of the analysis set (per-protocol [PP] set, N = 34, shown first; full analysis [FA] set, N = 38); 95% CIs use the Wilson method. OR is the odds ratio per Centiloid unit from logistic regression adjusting for age, sex, education, APOE ε4, and antidepressant use. "—" denotes models not estimable because of zero or near-zero events. No logistic model reached significance. Response categories are descriptive given the single-arm design.

Supplementary Table S3. *Amyloid prediction of treatment-related change across all 31 outcomes (covariate-adjusted, antidepressant use included): strongest regional amyloid indicator and the Centiloid model per outcome.*

Outcome (change)	Set	Strongest amyloid region	β (strongest)	p (strongest)	β (Centiloid)	p (Centiloid)	R^2
SCD-Q change, V1–V2	PP	Precuneus SUVR (R)	0.223	.207	0.279	.172	.149
	FA	Striatum SUVR (L)	−0.078	.561	0.158	.458	.082
GDS change, V1–V2	PP	Striatum SUVR (L)	−0.256	.228	−0.190	.494	.171
	FA	Striatum SUVR (L)	−0.312	.084	−0.222	.400	.148
ISI change, V1–V2	PP	Precuneus SUVR (R)	0.373	.057	0.137	.524	.122
	FA	Precuneus SUVR (R)	0.347	.048	0.146	.438	.146
MoCA change, V1–V2	PP	Striatum SUVR (L)	0.137	.388	−0.032	.845	.137
	FA	Striatum SUVR (L)	0.141	.281	−0.036	.812	.139
SCD-Q change, V1–V3	PP	Frontal SUVR (R)	0.150	.401	0.243	.210	.117
	FA	Frontal SUVR (R)	0.081	.628	0.189	.312	.106
GDS change, V1–V3	PP	Frontal SUVR (L)	0.227	.041	0.259	.041	.358
	FA	Precuneus SUVR (L)	0.138	.149	0.193	.071	.307
ISI change, V1–V3	PP	Precuneus SUVR (R)	0.355	.081	0.259	.165	.108
	FA	Precuneus SUVR (R)	0.310	.074	0.246	.172	.107
MoCA change, V1–V3	PP	Cingulate SUVR (R)	−0.356	.014	−0.423	<.001	.560
	FA	Cingulate SUVR (R)	−0.368	.018	−0.448	<.001	.520
MMSE change, V1–V3	PP	Cingulate SUVR (R)	0.285	.082	0.311	.043	.234
	FA	Cingulate SUVR (R)	0.284	.072	0.316	.038	.245
CERAD-K total 1 change, V1–V3	PP	Temporal SUVR (R)	0.194	.156	0.157	.366	.186
	FA	Frontal SUVR (R)	0.222	.121	0.186	.243	.147
CERAD-K total 2 change, V1–V3	PP	Temporal SUVR (R)	0.196	.263	0.104	.653	.113
	FA	Temporal SUVR (R)	0.200	.205	0.147	.478	.073
Time orientation change, V1–V3	PP	Cingulate SUVR (R)	0.360	.369	0.371	.317	.131
	FA	Cingulate SUVR (R)	0.351	.376	0.369	.317	.127
Place orientation change, V1–V3	PP	Precuneus SUVR (R)	0.110	.521	0.166	.351	.056
	FA	Striatum SUVR (L)	−0.075	.417	0.099	.467	.017
Attention change, V1–V3	PP	Temporal SUVR (R)	0.097	.198	0.016	.779	.167
	FA	Temporal SUVR (R)	0.104	.178	0.031	.607	.168
Boston Naming Test change, V1–V3	PP	Temporal SUVR	0.123	.451	−0.080	.680	.205
	FA	Frontal SUVR (L)	0.105	.539	−0.042	.805	.214
Verbal fluency change, V1–V3	PP	Temporal SUVR (R)	0.167	.415	0.112	.647	.128
	FA	Temporal SUVR (R)	0.157	.391	0.129	.562	.110
Word memory change, V1–V3	PP	Striatum SUVR (L)	0.149	.297	0.048	.786	.304
	FA	Striatum SUVR (L)	0.164	.210	0.088	.607	.273
Word recall change, V1–V3	PP	Precuneus SUVR (R)	−0.237	.032	−0.124	.233	.144
	FA	Precuneus SUVR (R)	−0.109	.331	−0.058	.605	.092
Word recognition change, V1–V3	PP	Cingulate SUVR (L)	0.431	.056	0.234	.315	.179
	FA	Cingulate SUVR (L)	0.332	.095	0.181	.397	.144
Construction recall change, V1–V3	PP	Frontal SUVR (L)	−0.110	.637	−0.118	.651	.163
	FA	Temporal SUVR (R)	0.066	.701	−0.066	.767	.107
Construction praxis change, V1–V3	PP	Cingulate SUVR (L)	0.089	.590	0.098	.492	.320
	FA	Cingulate SUVR (L)	0.091	.525	0.105	.411	.306
Trail Making Test A change, V1–V3	PP	Striatum SUVR (L)	−0.211	.306	−0.111	.556	.156
	FA	Cingulate SUVR (R)	−0.145	.404	−0.066	.686	.131
Trail Making Test B change, V1–V3	PP	Precuneus SUVR (R)	0.352	.029	0.253	.173	.177
	FA	Precuneus SUVR (R)	0.201	.200	0.177	.336	.116
Stroop color–word change, V1–V3	PP	Temporal SUVR (R)	−0.183	.207	0.070	.664	.171
	FA	Precuneus SUVR (R)	−0.097	.448	0.053	.701	.189
FAB similarities change, V1–V3	PP	Precuneus SUVR (R)	0.442	.041	0.517	.017	.322
	FA	Precuneus SUVR (L)	0.364	.093	0.434	.037	.246

FAB phonemic fluency change, V1–V3	PP	Cingulate SUVR (R)	−0.443	.010	−0.404	.013	.294
	FA	Cingulate SUVR (R)	−0.449	.002	−0.402	.006	.249
FAB motor series change, V1–V3	PP	Cingulate SUVR (R)	0.391	.276	0.356	.295	.191
	FA	Cingulate SUVR (R)	0.306	.337	0.287	.337	.199
FAB conflicting instructions change, V1–V3	PP	Temporal SUVR (R)	0.119	.347	−0.069	.485	.131
	FA	Cingulate SUVR (L)	−0.066	.622	−0.059	.451	.151
FAB go/no-go change, V1–V3	PP	Temporal SUVR (L)	0.210	.264	−0.018	.915	.207
	FA	Temporal SUVR (L)	0.241	.174	0.018	.915	.192
FAB prehension behavior change, V1–V3	PP	—	—	—	—	—	—
	FA	—	—	—	—	—	—
FAB total change, V1–V3	PP	Striatum SUVR (R)	0.138	.492	0.016	.939	.139
	FA	Temporal SUVR (L)	0.070	.681	−0.003	.988	.154

Note. For each outcome, the change score was regressed separately on each of 22 amyloid indicators (Centiloid plus 21 regional SUVRs), adjusting for age, sex, education, APOE ε4, and antidepressant use (per-protocol [PP] set, N = 34, shown first; full analysis [FA] set, N = 38). The strongest indicator has the smallest robust p value among the 21 regional SUVRs. β is standardized; heteroskedasticity-robust standard errors were used. FAB prehension had no variance in change and could not be estimated. Because many uncorrected tests were run, these results are exploratory and hypothesis-generating.

Supplementary Table S4

Benjamini–Hochberg False Discovery Rate Correction for the Exploratory Amyloid Analyses (31 Change Outcomes × 22 Amyloid Indicators)

Panel A. Summary by analysis set

**Analysis set**	**Models**	**Estimable**	**Nominal p < .05**	**Expected by chance**	**Survived BH**	**Minimum q**
Full analysis (N = 38)	682	660	31	~33	0	.120
Per-protocol (N = 34)	682	660	32	~33	0	.061

Panel B. Biologically motivated secondary family: Centiloid scale across the 31 outcomes

**Outcome (change)**	**Set**	**β**	**p**	**BH q**	**Survives**
MoCA	FA	−0.448	.0002	.005	Yes
MoCA	PP	−0.423	.0001	.003	Yes
FAB phonemic fluency	FA	−0.402	.0057	.088	No
FAB phonemic fluency	PP	−0.404	.0128	.173	No
GDS	FA	0.193	.0709	.440	No
GDS	PP	0.259	.0407	.267	No
MMSE	FA	0.316	.0381	.295	No
MMSE	PP	0.311	.0431	.267	No
FAB similarities	FA	0.434	.0367	.295	No
FAB similarities	PP	0.516	.0168	.173	No
Remaining 26 outcomes	FA, PP		p ≥ .165	q ≥ .672	No

Panel C. Phonemic fluency across the 22 amyloid indicators

**Set**	**β range**	**p range**	**BH q range**	**Indicators surviving**
FA	−0.454 to −0.317	.0025 to .0287	.017 to .029	22 of 22
PP	−0.444 to −0.282	.0098 to .1003	.069 to .100	0 of 22

Panel D. Associations surviving correction within each amyloid-indicator family (31 outcomes)

Set	Amyloid indicator	Outcome	β	p	BH q
FA	Centiloid scale	MoCA	−0.448	.0002	.005
PP	Centiloid scale	MoCA	−0.423	.0001	.003

Note. FA = full analysis set; PP = per-protocol set. All models were adjusted for age, sex, education, APOE ε4 carrier status, and antidepressant use, with robust standard errors. Twenty-two models per set were not estimable because FAB prehension change had zero variance, leaving 660 estimable models per set. No association survived Benjamini–Hochberg correction when all 660 estimable models were treated as a single global family. The number of nominally significant results was close to the rough number expected at $\alpha = .05$, although this benchmark is approximate because outcomes and amyloid indicators are correlated. The 22 amyloid indicators derive from a single PET scan and are highly correlated; consistency across indicators in Panel C therefore reflects robustness to amyloid quantification rather than independent replication. The Centiloid-only family is presented as a biologically motivated secondary analysis, not as a preregistered confirmatory family. All findings remain exploratory and hypothesis-generating.

Interpretation. These analyses were conducted to quantify rather than minimize the multiplicity inherent in the exploratory amyloid models. When all 660 estimable models were treated as a single global family, no association survived correction (minimum q = .120 in the full analysis set and .061 in the per-protocol set), and the nominally significant count was close to a rough chance benchmark. This result argues against a broad, diffuse, or convergent amyloid-related pattern across cognition. It does not, however, require treating every structured secondary result as equally uninformative. In the biologically motivated Centiloid-only family, the inverse association with MoCA change survived correction in both analysis sets (q = .005 and .003), whereas the other Centiloid associations did not.

The appropriate interpretation is therefore hierarchical. The Centiloid–MoCA association is the only signal that remained stable across the full-analysis and per-protocol sets within the same focused family, making it the most defensible hypothesis generated by the study. The phonemic-fluency association was less stable: it survived correction across the 22 highly correlated amyloid indicators in the full analysis set but not in the per-protocol set. Most other cognitive outcomes showed no association, arguing against a generalized cognitive effect. These findings do not establish amyloid as a biomarker or prove that the association is stimulation-specific, but neither are they best summarized as an undifferentiated collection of chance results. They identify one focused global-cognition hypothesis that warrants confirmatory testing in an adequately powered, sham-controlled study with prespecified outcomes.
